# All Roads Lead to Rome: Exploring Human Migration to the Eternal City through Biochemistry of Skeletons from Two Imperial-Era Cemeteries (1st-3rd c AD)

**DOI:** 10.1371/journal.pone.0147585

**Published:** 2016-02-10

**Authors:** Kristina Killgrove, Janet Montgomery

**Affiliations:** 1 Department of Anthropology, University of West Florida, Pensacola, FL, United States of America; 2 Department of Archaeology, Durham University, Durham, United Kingdom; Museo Nazionale Preistorico Etnografico ‘L. Pigorini’, ITALY

## Abstract

Migration within the Roman Empire occurred at multiple scales and was engaged in both voluntarily and involuntarily. Because of the lengthy tradition of classical studies, bioarchaeological analyses must be fully contextualized within the bounds of history, material culture, and epigraphy. In order to assess migration to Rome within an updated contextual framework, strontium isotope analysis was performed on 105 individuals from two cemeteries associated with Imperial Rome—Casal Bertone and Castellaccio Europarco—and oxygen and carbon isotope analyses were performed on a subset of 55 individuals. Statistical analysis and comparisons with expected local ranges found several outliers who likely immigrated to Rome from elsewhere. Demographics of the immigrants show men and children migrated, and a comparison of carbon isotopes from teeth and bone samples suggests the immigrants may have significantly changed their diet. These data represent the first physical evidence of individual migrants to Imperial Rome. This case study demonstrates the importance of employing bioarchaeology to generate a deeper understanding of a complex ancient urban center.

## Introduction

The Medieval aphorism *Mille viae ducunt homines per saecula Romam* (*Liber Parabolarum* 591), translated as “All roads lead to Rome,” was likely a reference to the *miliarium aureum*, which Plutarch (*Life of Galba*, 24.2) described as a gilded column in the Forum Romanum where every road in the Italian peninsula ended. Early in his principate, Augustus set up this monument and inscribed distances between Rome and other cities in the Empire [1]. As the literal center of the Empire, Rome beckoned people into its walls with the promise of bread and circuses, jobs and culture, but the effects that visitors had on various aspects of society is still a nascent research topic [2].

Roman demographers have synthesized the historical, epigraphical, and archaeological evidence of migrants to the capital and have contributed to big-picture questions of migration, fertility, and mortality [3–5]. In the absence of a quantifiable data source such as a census, however, smaller scale migration often becomes a footnote [6–8]. Without data on those specific individuals and groups who made the journey to Rome either by force or by choice, of their biological and cultural characteristics, their effects on the Roman population, or their experiences negotiating a new geographical space, we stand to miss out on a plethora of information on what it was like to be an immigrant to Imperial Rome.

Human skeletal remains from cemeteries in Rome are an obvious new line of evidence, and biochemical analysis of tissue can yield information about migration in the absence of epigraphical and archaeological indications of foreigners in Rome. Yet bioarchaeology in Rome is still a young and underfunded field of research, in spite of the thousands of skeletons that exist from the Republican and Imperial periods [9–18]. Investigating the biological remains of the ancient Romans is imperative for a deeper understanding of those individuals who are not well-represented by records that are biased towards wealthy, literate, elite males: namely, women, children, the lower classes, slaves, and free immigrants.

In this paper, we demonstrate through a case study of two Imperial-era cemeteries in Rome both the limitations and the potential of biochemical analyses for deepening our understanding of the phenomenon of migration within a complex ancient society. Namely, this study begins to answer the questions: (a) Who migrated to Rome? (b) From where? and (c) What was their experience at their destination? When Rome as the center of an empire is approached anthropologically using all available data sources, migrants become actors and slaves become diasporic individuals, and the effects of population interaction on both locals and foreigners can be questioned in a novel way.

### Imperial Rome and Its Immigrants

By the beginning of the Empire, the Italian peninsula was already quite heterogeneous in its population, and there were no stark cultural or linguistic borders between Italic peoples. Augustus’ *Pax Romana* created a contiguous geopolitical area in which peoples of the Empire could move freely if they had the finances necessary to undertake such a move. By the early 1st century AD, the 14 km^2^ city of Rome (*urbs*) had a large population living within its walls and in the periurban area (*suburbium*) just outside it [3, 19–23]. Precise population numbers are unclear, owing to changing Roman census and recording practices as well as to contemporary extrapolation of ancient estimates, but scholars tend to agree that Imperial Rome had a dense population of up to one million people and at least moderate migration rates that helped to combat high mortality rates [20, 24–26].

While voluntary immigrants likely represented about 5% of the population of Imperial Rome [2, 4, 27], slaves accounted for up to 40% [28]. Many of these slaves were *vernae*, locally-born offspring of a slave mother, but others would have come to Rome from other areas of Italy or from far-flung regions of the Empire. The populations of both voluntary and compulsory migrants, as well as the geographic areas from which they hailed, changed generation to generation [2, 25, 28, 29], but there is no complete Imperial-era census for the city of Rome [23, 30].

The current data sets on migration to Imperial Rome therefore include the historical record, archaeological remains, and epigraphical inscriptions on tombstones. None of these lines of evidence is, of course, perfectly reflective of the experiences of all immigrants to Rome. The historical record is notoriously biased towards elite men with money, power, and literacy [31] and may not represent accurately the lives of the average voluntary immigrant or slave. At Rome, slaves tended to be integrated into the household, so we cannot expect to find clear archaeological evidence of slavery in the same way as, for example, in the Southern U.S., with separate quarters or special pottery assemblages [32–34]. The epigraphic record is perhaps the most useful at identifying individual migrants, but only when a person is specifically commemorated as a foreigner [2, 25].

Biochemical analysis of human skeletal remains to identify ancient migrants has been undertaken for almost two decades through the use of strontium and oxygen isotope analysis of dental enamel [35–45], but this method has not been applied to skeletons from Imperial Rome before.

The challenge with Rome, one of the most complex societies of the past, lies in integrating osteological remains with textual sources and archaeological data to create a contextualized, historical bioarchaeology [46]. An historically-contextualized bioarchaeological approach to migration to Rome presented in this case study allows for the investigation of questions like: (a) Who immigrated to Rome? (b) Where did they come from? (c) Did immigrants acculturate or change their culturally-influenced behaviors after their arrival? The first step in answering these questions is to find evidence of foreigners in Rome using the variation in isotopes of strontium and oxygen in ancient Roman bodies.

### Sr and O Isotopes in Ancient Italy

Strontium passes essentially unchanged into the biosphere from the weathering of rocks, and most of the strontium in the body is present in the skeleton because, as another divalent alkaline earth metal, strontium follows calcium in most environmental and biological processes [47, 48]. A human’s ^87^Sr/^86^Sr isotope ratio from dental enamel therefore reflects both diet and, to a lesser extent, drinking water. In ancient communities that farmed and obtained water locally, human ^87^Sr/^86^Sr isotope ratios should reflect the underlying geology. Oxygen isotopes, on the other hand, are related to environmental and meteoric water, and the values change based on factors such as latitude, rainfall, elevation, humidity, temperature, and distance from the coast [49, 50]. A human’s *δ*^18^O phosphate and carbonate ratios are mostly related to the isotope composition of drinking water [51–54]. After taking into account metabolic fractionation between enamel and body water, oxygen isotope ratios can identify individuals who consumed local or nonlocal water while their tissues were forming [53–55]. Individuals local to an area will have strontium and/or oxygen isotope ratios in line with the water, soil, and rocks in the region, while nonlocal individuals’ ratios will be anomalous compared to the local value. Comparing the isotope ratios in human tissue with geology and climatology data, it may also be possible to propose the geographical location of the homelands of nonlocal individuals [56].

Strontium and oxygen isotope analyses have been frequently used in tandem to study migration in past populations [37, 57, 58], and recent studies are including DNA alongside isotopic evidence [59, 60]. Previous studies of migration and mobility within the Roman Empire using strontium isotopes have been undertaken in areas such as Late Roman Bavaria [45], Greece [61], and northeastern Africa [62], but Roman Britain is the best studied so far, with most studies utilizing multiple isotopes (Sr, O, C, and/or N) [63–71]. Italy does not have much published strontium data, with the only human results coming from the author’s previous publication from Republican Rome (3rd to 1st century BC) [72], from Iron Age Monte Bibele (4th to 3rd century BC) in northern Italy [73], and from Neolithic Apulia (6th millennium BC) in southeastern Italy [74]. The strontium results presented here therefore represent only the fourth set of human results from ancient Italy and the first for the Imperial period; they also serve both to answer questions about mobility in the Roman Empire and to generate baseline and comparative data to aid future studies of migration. Few oxygen isotope studies have been done to investigate migration in the Roman Empire. In addition to the Romano-British studies referenced above, Imperial-era oxygen isotope studies have been accomplished in Egypt [75] and at Portus Romae, Italy [76].

**Fig 1:**
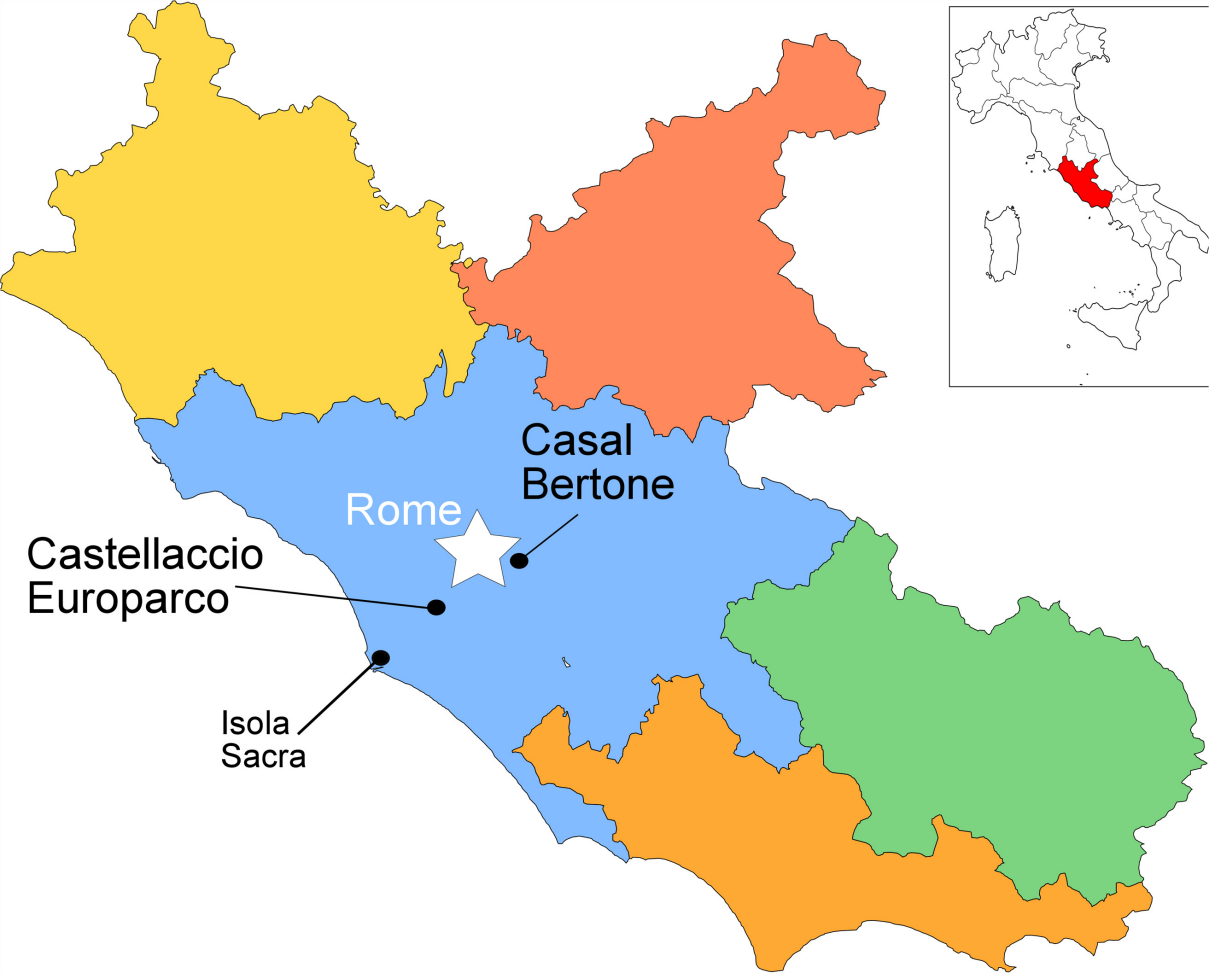
Imperial Roman and Suburban Cemeteries. Public domain map via Wikimedia Commons, modified to include archaeological sites.

Prowse and colleagues [76] have produced the only human oxygen isotope data for central Italy to date, and these data therefore comprise a comparative data set for the oxygen isotope ratios obtained in this study. The 1st to 2nd century AD cemetery of Isola Sacra, from which Prowse and colleagues’ dental remains came, was associated with Portus Romae, a port city located about 25 km southwest of Rome on the Tyrrhenian Sea (Fig 1). Prowse and colleagues [76] analyzed first and third molars from individuals buried in the Isola Sacra cemetery in order to understand patterns of migration within the population of Portus Romae. They interpreted the continuum of oxygen isotope ratios (see below) as evidence of people coming to Portus Romae from nearby locales [76]. Additionally, they found statistically significant differences between the first and third molars of numerous individuals, which they interpreted as evidence of movement during childhood [76]. While more of the third molars had higher oxygen isotope ratios, for a large proportion of the sample, the first molars had higher oxygen isotope ratios. It is possible that the higher first molar/lower third molar values are related to changes to oxygen isotopes following cessation of breastfeeding rather than migration, although this is not addressed in their study. Regardless, their analysis demonstrated that males, females, and children were mobile during the Roman Empire, an important finding that contributed new data to the question of the demographics of immigrants to Portus Romae.

The present case study builds on Prowse and colleagues’ data set and adds both strontium and oxygen isotope data from Imperial Rome itself, revealing a number of individual immigrants to Rome. Results of isotope analyses from two cemeteries associated with Imperial Rome are presented below, followed by a critical discussion of integrating biochemical data into the research tradition of classical studies and by suggestions for future directions in migration studies in historical bioarchaeology.

## Materials and Methods

The collections that were used for this analysis are overseen by the Servizio di Antropologia, Soprintendenza Speciale per i Beni Archeologici di Roma. Permission to extract, transport, and test the samples was also provided by the Servizio di Antropologia on behalf of the Ministero per i Beni e le Attività Culturali, in compliance with all relevant regulations concerning human remains from ancient Italy. All human remains from these two sites were first analyzed osteologically by KK, who also took the samples and performed the Sr analysis, while JM performed the O analysis. The unused samples have since been returned to Italy.

### Sites

The two cemeteries that provided human skeletal material for this analysis are located in the *suburbium* of Rome (Fig 1), as almost all burials were relegated outside the city walls for health and religious purposes [77]. It is therefore not known whether the people buried in these cemeteries lived within the city walls or outside in the *suburbium*; most likely, there is a mixture of both.

The Casal Bertone cemetery is located roughly 1.5 km east of the Aurelian walls of Rome along the ancient *via Praenestina*. Salvage excavations occurred between 2000-03, and archaeologists date the cemetery to the 2nd-3rd centuries AD [78, 79]. A necropolis component with simple inhumations in pits and in *cappuccina*-style graves [77] slightly predates the above-ground mausoleum structure, which included niches for single and multiple burial.

The Castellaccio Europarco cemetery was excavated between 2003-07 [16, 80, 81], about 12 km south of the Roman *urbs* near a retaining wall of the ancient *via Laurentina*. The cemetery is less structured than Casal Bertone, with burials largely of the pit and *cappuccina* varieties arranged haphazardly. Castellaccio Europarco was used as a burial area for centuries, and artifacts and building styles helped archaeologists separate the main use periods into two Republican-era burial phases (4th-3rd centuries BC and 2nd-1st centuries BC; see [72] and below for isotope data from this period) and one Imperial-era burial phase (1st-2nd centuries AD).

Burials at both cemeteries by and large lack grave goods, and the burial style is without elaboration or grave markers, meaning there is no clear indication who was buried in these cemeteries. The northern portion of the Casal Bertone complex, however, included two funeral buildings with *cippi* (pillars) inscribed with the names of slaves or freedmen of Greek ancestry: L. Cincius Nasta and C. Ateius Epaphra (*Corpus Inscriptionum Latinarum* VI 37587 and VI 37576). Although these funeral buildings did not contribute material to this study, it is reasonable to assume that individuals born outside of Rome were buried at Casal Bertone.

### Demographics

The demographics of the individuals assessed in this study (n = 105) are provided in Tables 1 and 2 below. Age-at-death was assessed based on the pubic symphysis [82–84], the auricular surface [85], cranial suture closure [86], dental development [87–91], and epiphyseal closure [92]. Subadults were classified per [92] into the categories of Infant (I: 0-12 months), Young Child (YC: 1-6), Older Child (OC: 7-12), and Adolescent (AD: 12-20). Adults were classified per [93] into the categories of Young Adult (YA: 20-35), Middle Adult (MA: 35-50), and Old Adult (OA: 50+). Sex of adults and older adolescents (age 16-20) was estimated based on pelvic morphology [93, 94] and cranial features [95].

**Table 1:** Chemical Analysis Results from Castellaccio Europarco.

Skeleton	Sex	Age	^87^Sr/^86^Sr (enamel)	*δ*^18^O_*carb*_ (enamel) ‰ VSMOW	*δ*^13^C_*ap*_ (enamel) ‰ VPDB	*δ*^13^C_ap_* (bone) ‰ VPDB
ET16	I	YC	0.70966			
ET17	I	OC	0.70883			
ET18	F	YA	0.70862	26.3	−12.5	−10.4
ET20	M	MA	0.70963	25.3	−4.0	−8.6
ET22B	M	YA	0.70840			
ET27	PM	AD	0.70954	28.2	−12.1	−10.2
ET31	I	YC	0.70985	26.5	−12.4	−10.9
ET33	PM	MA	0.70878			
ET36	I	OC	0.70957	24.9	−12.8	−10.1
ET37	I	OC	0.70957			
ET38	M	MA	0.71193	25.7	−7.6	−9.8
ET40	F	MA	0.70918			
ET42	PM	Adult	0.70925	26.7	−13.8	
ET43	M	MA	0.70813			
ET44	M	AD	0.71015	26.7	−13.5	−9.3
ET45	M	AD	0.70940	25.7	−12.6	
ET51	PM	MA	0.70934			
ET52	PM	YA	0.70872			
ET58	F	MA	0.70916	25.4	−11.7	−10.8
ET63	I	OC	0.70935			
ET67	I	OC	0.70917	27.9	−12.5	−12.2
ET68	F	MA	0.70875	27.2	−12.5	
ET69	M	YA	0.70862	26.9	−13.1	−10.7
ET72	M	MA	0.70100	27.1	−12.1	−10.9
ET76	PM	AD	0.71047			
ET103	PM	MA	0.70911			
Burial	Genus	Tooth				
ET20	*Sus*	molar	0.71031			

Age Categories: I = infant; YC = young child; OC = older child; AD = adolescent; YA = younger adult; MA = middle adult; OA = older adult. Sex Categories: M = male; PM = probable male; I = indeterminate; PF = probable female; F = female.

* Data published in [98].

**Table 2:** Chemical Analysis Results from Casal Bertone.

Skeleton	Sex	Age	^87^Sr/^86^Sr (enamel)	*δ*^18^O_*carb*_ (enamel) ‰ VSMOW	*δ*^13^C_*ap*_ (enamel) ‰ VSMOW	*δ*^13^C_ap_* (bone) ‰ VPDB
F1A	PF	AD	0.70930	25.7	−12.0	−12.9
F1B	M	MA	0.70904			
F1C	F	OA	0.70962			
F1D	PM	AD	0.70879			
F3C	M	MA	0.70835	26.1	−12.5	−12.9
F4A	I	OC	0.70985			
F4B	F	OA	0.70982	25.5	−13.2	−13.3
F4C	PM	AD	0.70919			
F5A	M	YA	0.70995	25.0	−11.7	−11.3
F6E	F	OA	0.70894	26.6	−12.4	−13.2
F7B	M	AD	0.70946	27.8	−12.3	−12.6
F9B	I	YC	0.70977			
F9C	PF	YA	0.70921			
F10B	I	OC	0.70928			
F10C	I	OC	0.70825	27.4	−10.4	−12.3
F10D	I	OC	0.70989	27.4	−12.0	−12.7
F11A	F	MA	0.70971	26.7	−13.0	
F11B	M	MA	0.70901			
F11C	I	AD	0.70958			
F12A	M	YA	0.70930	26.5	−14.2	−12.8
F13A	I	OC	0.70943			
F13C	F	MA	0.70946	26.0	−12.5	−12.8
T7	M	MA	0.70940	26.3	−12.5	−11.8
T8	I	OC	0.71065	25.4	−10.9	
T9	I	YC	0.70930			
T10	M	MA	0.70957	26.1	−12.1	−13.8
T11	I	AD	0.70933			
T12	M	AD	0.70955			
T13	PM	OA	0.70849	26.4	−12.0	−12.7
T14	M	YA	0.70899	25.9	−13.2	
T15	PM	MA	0.71398	25.6	−12.3	
T18	PM	YA	0.70949	25.8	−12.8	−13.7
T19	M	MA	0.70915	24.9	−10.9	
T20	I	OC	0.70909	26.0	−13.5	−12.9
T21	M	AD	0.70881	26.6	−13.5	−11.3
T22	M	YA	0.70907			
T23	M	YA	0.70842	26.4	−11.3	−11.4
T24	M	OA	0.70735	24.4	−10.9	−12.7
T26	PM	Adult	0.70918			
T28	F	OA	0.70853	25.3	−11.8	−11.7
T29	I	YC	0.70933	25.8	−12.4	−12.2
T30	PF	MA	0.70922	26.6	−12.3	−12.1
T31	M	MA	0.70918			
T32	I	AD	0.70918	27.4	−12.4	
T33	M	MA	0.70816	27.6	−12.7	
T34	M	MA	0.70907	24.8	−12.2	−11.7
T35	PM	AD	0.70946			
T36	I	AD	0.70719	28.5	−6.8	−10.4
T37	PM	YA	0.70918			
T38	PF	MA	0.70932			
T39	PF	AD	0.70821	28.8	−10.9	−11.7
T41	PF	AD	0.70917			
T42	F	YA	0.70928	27.2	−12.6	−12.5
T45	I	AD	0.70924			
T47	M	YA	0.70865			
T48	PF	Adult	0.70951			
T49	I	MA	0.70915			
T50	PF	YA	0.70931	26.6	−12.9	−12.2
T53	PM	YA	0.70850			
T55	I	YC	0.70893			
T56	I	AD	0.70951			
T59	M	Adult	0.70859			
T60B	I	YC	0.70859			
T62	I	OC	0.70916			
T66	PM	Adult	0.70873			
T67	PM	MA	0.70835			
T69A	M	MA	0.71009			
T70	I	OC	0.70898	28.9	−12.6	−12.2
T71	I	YC	0.70904	25.7	−13.1	−12.8
T72	I	OC	0.70791	25.6	−11.9	
T73	M	MA	0.70913			
T75	I	AD	0.70874			
T76	PM	MA	0.70942	26.4	−12.4	−12.0
T77	PM	MA	0.70914			
T80	I	AD	0.70906	27.1	−14.8	−13.1
T81	M	YA	0.70885	25.0	−12.9	
T82A	F	MA	0.70862	24.3	−12.8	−12.9
T83B	M	AD	0.70878	26.1	−13.4	−12.6
T84	I	AD	0.70890			
Burial	Genus	Tooth				
US31	*Sus*	molar	0.70933			

Samples that start with F are from the mausoleum, and those that start with T are from the necropolis. Age Categories: I = infant; YC = young child; OC = older child; AD = adolescent; YA = younger adult; MA = middle adult; OA = older adult. Sex Categories: M = male; PM = probable male; I = indeterminate; PF = probable female; F = female.

* Data published in [98].

### Sample Selection

The total number of individuals examined osteologically from the Imperial phases of Casal Bertone and Castellaccio Europarco is 189 [96]. In order to create as robust a sample as possible, the enamel from every individual with an intact first molar was assessed for strontium (^87^Sr/^86^Sr). Three individuals from Castellaccio Europaro had pathological first molars (ET33, ET37, ET43), so a canine was substituted, as the timing of its formation is similar to that of the first molar [90]. The resulting strontium sample size is 105, with 26 individuals from Castellaccio Europarco, 22 from the Casal Bertone mausoleum, and 57 from the Casal Bertone necropolis. Although these strontium samples were published in the aggregate in [97], the data are presented individually and fully contextualized here.

A demographically stratified sample of this population was also analyzed for oxygen isotopes (*δ*^18^O) as well as carbon isotopes from the enamel carbonate fraction of the first molars (*δ*^13^C_*ap*_), resulting in a sample population consisting of 14 individuals from Castellaccio Europaro, 11 individuals from the Casal Bertone mausoleum, and 30 individuals from the Casal Bertone necropolis. Carbon and nitrogen isotope data from bone samples from these populations were previously published [98], and those *δ*^13^C_*ap*_ palaeodietary data are reproduced in the tables. In addition to the human samples, two *Sus* teeth recovered during archaeological excavation were processed for strontium according to the methodologies described below.

### Biochemical Procedures

Strontium isotope analysis was accomplished at the Isotope Geochemistry Laboratory at the University of North Carolina at Chapel Hill by the author with the assistance of Dr. Drew Coleman. Each tooth was cleaned by surface abrasion. Between 5 to 10 mg of dental enamel was extracted using a Brasseler hand-held dental drill fitted with a 0.3 mm round tungsten carbide bit, weighed on a Sartorius microbalance, and stored in 5 mL Savillex vials with deionized water prior to processing. Strontium was extracted by dissolving the powdered enamel in 500 *μ*L of 7M HNO_3_, then evaporating and redissolving it in 500 *μ*L of 3.5M HNO_3_. Sr-Spec^™^ columns were cleaned and loaded with 50 to 100 *μ*L of EiChrom SR-B100-S resin, and the enamel sample was centrifuged. The sample was loaded by pipette from the centrifuge vial and subjected to dropwise and bulk sample rinses with HNO_3_. Strontium was eluted into a clean Savillex vial with deionized water, 25 *μ*L of H_3_PO_4_ was added, and the water was allowed to evaporate on a hotplate. The sample was redissolved with 2 *μ*L of TaCl_5_. Half of the strontium was loaded onto a rhenium filament, and the ^87^Sr/^86^Sr isotope ratio was measured on a fully automated VG Micromass Sector 54 TIMS spectrometer in reference to standard NBS-987, which has a ratio of 0.710270 ± 0.000014 (absolute, 2*σ*), based on replicate analyses of the standard run over the same period as the samples. The internal precision for individual strontium runs was ± 0.000008 to 0.000013 (absolute, 2*σ*) standard error based on 100 triple-dynamic cycles of data collection.

Analysis of the light isotopes of carbon and oxygen was performed at the Stable Isotope Facility at the University of Bradford. The outer layer of each tooth was cleaned using a diamond dental burr. A single enamel sample of around 15 mg was extracted from the thickest part of the tooth wall, rather than the earlier-forming occlusal region, following the procedure of [48]. Between individuals, the dental burr was cleaned with 4M HNO_3_, rinsed with deionized water, placed in an ultrasonic bath for five minutes, and swabbed with acetone.

The procedure for pre-treatment of enamel apatite is based on [99]. Each sample received 1.8 mL of NaOCl. Samples were rinsed with deionized water and centrifuged three times. 1.8 mL of 0.1M acetic acid was added, and the samples were again rinsed with deionized water and centrifuged three times. Samples were heat-dried overnight and freeze-dried before being weighed and loaded onto the IRMS. Rinsed and freeze-dried samples were weighed in duplicate and measured using a Finnigan Gasbench II connected to a Thermo Delta V Advantage continuous flow isotope ratio mass spectrometer. Enamel carbonate was reacted with anhydrous phosphoric acid at 70deg C to release CO_2_ gas from which *δ*^18^O_*VSMOW*_ and *δ*^13^C_*VPDB*_ were determined using a CO_2_ reference supply.

Data were normalized by means of a linear calibration equation derived from a plot of accepted versus measured values for two internal standards, Merck Spurapur CaCO_3_ and OES (ostrich egg shell), and the NBS19 international standard. Ratios of *δ*^18^O and *δ*^13^C are reported, respectively, per international standards Vienna Standard Mean Ocean Water (VSMOW) and Vienna Pee Dee Belemnite (VPDB). Analytical precision was determined using an internal enamel laboratory standard to be ± 0.1‰ (1 stdev) for *δ*^13^C and ± 0.2‰ (1 stdev) for *δ*^18^O.

## Results

### Demographics

The Castellaccio Europaro sample (n = 26) breaks down into 7 subadults (I, YC, and OC categories) and 19 adults and older adolescents (15 male and 4 female). Most of the adults (n = 10) fall into the Middle Adult (35-50) age category. There is an obvious underrepresentation of females in this cemetery population, but it is unclear if this is the result of burial practices, taphonomy, or another phenomenon. The Casal Bertone sample includes 25 subadults (I, YC, and OC categories plus younger AD) and 54 adults and older adolescents (37 male, 16 female, and 1 indeterminate sex). The Middle Adult age category is similarly the most populated (n = 22), but there was also a comparable number of Adolescents (n = 19). Unlike at Castellaccio Europarco, where the oldest individuals fell into the Middle Adult category, the Casal Bertone sample population includes 6 Older Adults (4 female and 2 male). The demographics of these two sites are different, possibly owing to taphonomy or burial practices, but it is clear that life expectancy was low for both non-elite populations.

### Chemical Analyses

Results of the chemical analyses are presented in Tables 1 and 2. These data include measurements from dental enamel of ^87^Sr/^86^Sr from all individuals in the sample (n = 105) and of *δ*^18^O and *δ*^13^C_*ap*_ from 55 individuals. Each table additionally includes *δ*^13^C_*ap*_ ratios from bone apatite previously published in [98]. All osteological and biochemical data for these two cemeteries can be found in [100].

Sample numbers are the same as tomb numbers assigned by the Soprintendenza Speciale per i Beni Archeologici di Roma, who excavated them; prefixed letter identifiers are ET for Castellaccio Europarco tombs, T for Casal Bertone tombs, and F for Casal Bertone mausoleum burials.

A series of statistical tests to look for hidden inter- and intrapopulation variation was done using the Mann-Whitney U statistic on pairs of sites, sex, and burial form. At the inter-site level, the mean strontium isotope ratio from Castellaccio Europarco (0.70935) is higher than the mean at Casal Bertone (0.70915), but a Mann-Whitney U test does not show statistical significance (U = 850.5; p = 0.19; n = 105). There was also no statistical difference between the mean oxygen isotope ratios at Castellaccio Europarco (26.5‰) and Casal Bertone (26.3‰) (U = 253.5; p = 0.5; n = 55).

At the intra-site level, comparisons can be made between the sexes. No statistically significant differences obtained between the sexes at Casal Bertone for strontium isotopes (U = 216; p = 0.12; n = 53) or oxygen isotopes (U = 82.5; p = 0.34; n = 30); there were too few individuals tested from Castellaccio Europarco to perform statistical tests based on sex.

The Casal Bertone sample includes individuals from a mausoleum context and a necropolis context. The means of the oxygen isotope ratios are not significantly different between burial types (U = 138.0; p = 0.43; n = 41). Strontium isotope ratios, however, are higher on average in the mausoleum sample (0.70933 ± 0.00046) compared to the slightly earlier necropolis (0.70908 ± 0.00011) at Casal Bertone, and the Mann-Whitney U test of the means is significant (U = 383.5; p = 0.008; n = 79). The Casal Bertone mausoleum sample has a much narrower range of values (0.70825–0.70995) than does the necropolis sample (0.70719–0.71398). The greater range of variation within the necropolis sample of Casal Bertone, as shown further below, is primarily owed to three individuals with strontium isotope ratios significantly different than the remainder of the population.

### Approximating Sr and O Isotope Ranges for the Roman *Suburbium*

Interpreting human oxygen and strontium isotope ratios in order to arrive at conclusions of migration and mobility requires an assessment of the local isotope range. The only published human strontium isotope data from the Roman *suburbium* are six data points from Republican-era Castellaccio Europarco [72], and Prowse and colleagues’ work at nearby Portus Romae [76] has produced the only oxygen isotope results in central Italy aside from the author’s four data points from Republican-era Castellaccio Europarco [72]. Due to the complexity of both the volcanic geology of Rome and the importation of drinking and irrigation water via aqueducts, local ranges must be conservatively estimated based on published data and on statistical analysis of the sample populations themselves.

#### Local Strontium Isotope Range and Results

**Fig 2:**
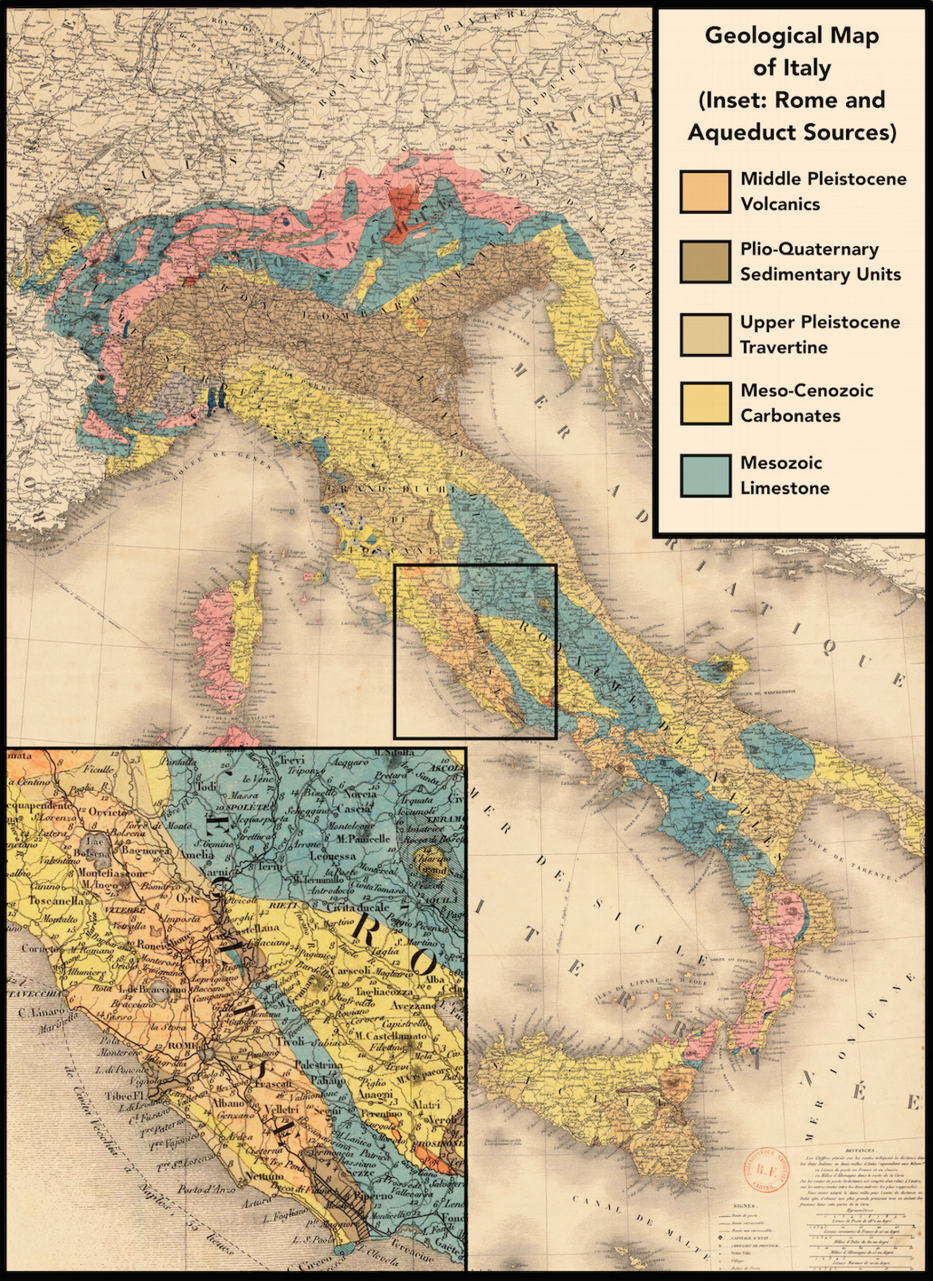
Geological Map of Rome. Public domain image of the geological map of Italy by H. de Collegno, 1844. Bibliothèque nationale de France, dèpartment Cartes et plans, GE DL 1844-126, modified to include an inset of Rome and a legend for the geology.

Rome is positioned along the Tiber River between two dormant volcanic complexes, the Colli Albani and the Monti Sabatini. The city is surrounded by Middle to Upper Pleistocene volcanic rock as well as Plio-Quaternary sedimentary units along the Tiber, which extend westward to the Tiber Delta and end at the Tyrrhenian Sea near Portus Romae. To the east of Rome are the Apennine Mountain foothills (Preapennines), composed of Meso-Cenozoic sandstones and limestones [101, 102]. A simplified map of the geology of this area is presented in Fig 2, while a full geological map of the area can be found in [103]. Although the geology of Rome may appear at first glance to be quite complex, the city and its surroundings consist primarily of geologically young rock.

Strontium isotope ratios from rocks, faunal remains, and human remains are available to characterize the local ^87^Sr/^86^Sr biosphere. The lavas surrounding Rome are variable in strontium isotope ratios, which may be the result of a petrogenetic transition between the Colli Albani magma and the more radiogenic Monti Sabatini magma [104]. Table 3 lists the published data points for the three major geographic areas from which Roman drinking and irrigation water would have come. The *urbs* and *suburbium* of Imperial Rome exist primarily within the Colli Albani, composed of Middle Pleistocene volcanic rock that ranges from 0.70900–0.71003 [105–107]. Isotope results from ancient faunal remains found in this geographic area fall within the range expected from animals living on pyroclastic volcanic geology: 0.70933–0.71031 [105, 108, 109].

**Table 3:** Strontium Isotope Ratios Around Rome.

Type of Data	Data Source	^87^Sr/^86^Sr Mean	^87^Sr/^86^Sr Range
Geology			
	Colli Albani [105, 107]	0.70965 (n = 7)	0.70900–0.71003
	Monti Sabatini [110]	0.71049 (n = 3)	0.70987–0.71116
	Monti Simbruini [111]	0.707837 (n = 9)	0.707413–0.708060
Fauna			
	*Elephas antiquus*	0.710007 (n = 19)	0.70985–0.71021
	Colli Albani—Casal de’ Pazzi [108]		
	*Elephas antiquus*	0.709911 (n = 20)	0.70980–0.71000
	Colli Albani—La Polledrara [108]		
	*Sus scrofa*	N/A (n = 2)	0.709326, 0.710313
	Colli Albani (Present Study)		
	*Cervus elaphus*	0.708674 (n = 6)	0.708325–0.708837
	Colli Albani/Monti Simbruini—Grotta Polesini [101]		
Humans			
	Castellaccio Europarco (Republican Era) [72]	0.70945 (n = 6)	0.70718–0.71013

A typical sedentary agricultural population in the past would have ingested strontium from two main sources: the geology from which the population procured their crops and the water that helped grow them [112]. Imperial Rome was not typical, however, as it was importing roughly 115 million gallons of water per day for both irrigation and drinking water, according to the figures of Frontinus, commissioner of the aqueducts in the 1st century AD [113]. Three main sources of water flowed into Rome through nine aqueducts: 1) the Aniene River and springs in the Monti Simbruini to the east; 2) springs in the Colli Albani; and 3) the lake area of Monti Sabatini to the north [114] (see Fig 2). Potable water was meted out within the city to both private consumers and to public works (fountains, baths, basins, etc.) and was available to everyone: women as well as men, slaves as well as free citizens [114]. Individual *castella* in Rome were distribution tanks or cisterns that were usually fed by the water of one aqueduct [113], but multiple *castella* with multiple water sources could be found in the same location. Water was also piped into the *suburbium* to supply baths, fountains, and industries, and many suburban dwellers illegally tapped the aqueducts along their route [113, 39].

There are significant outcrops of Upper Pleistocene travertine near Rome at Tivoli, and there is abundant limestone in the Apennine foothills of the Monti Simbruini that dominates the geology of numerous freshwater springs that fed the three eastern aqueducts [111, 115]. Measured strontium isotope ratios from rock in the Monti Simbruini are lower than the volcanic geology of the Colli Albani and range from 0.70741–0.70806 [111]. Ancient faunal remains from the site of Grotta Polesini, located roughly between the volcanic rock of the Colli Albani and the predominantly Pliocene-Lower Pleistocene limestone that characterizes the Monti Simbruini, fall as expected between the higher and lower strontium isotope ranges: 0.70833–0.70884 [101]. Two aqueducts with sources in the Monti Sabatini may also have contributed to human strontium isotope ratios, as this water was primarily used for irrigation. The few published data points for the Sabatini volcanic complex give a range of 0.70987–0.71116, slightly higher than the Colli Albani. Rome is not far from the Tyrrhenian Sea, which has a strontium isotope ratio of 0.7092 [116]. A previous isotopic study of carbon and nitrogen isotopes on a subset of the same populations [98] showed seafood was not a primary part of the diet; rather, C_3_ grains (likely wheat) and terrestrial meat were. Although seawater was not used for growing crops and the Romans were not likely eating seaweed, the strontium isotope ratio of the sea cannot be ruled out as an end-member contributing to human strontim isotope values.

Individuals who lived in or frequented the city and suburbs of Rome thus had access to drinking water that came from two distinctly different geological sources. More importantly, aqueduct water was used for irrigation; per Frontinus, roughly 25% of the water that came in from the Monti Simbruini and all of the water imported from the Monti Sabatini was used either for Augustus’ *naumachia* (a large basin in which mock naval battles were held) or for irrigation. Therefore, a child who grew up in Rome could have obtained drinking water from the Colli Albani or Monti Simbruini and could have eaten food (e.g., wheat, fruit, and vegetables; see [98]) grown with water from the Colli Albani or with water imported from the Monti Sabatini or the Monti Simbruini.

**Fig 3:**
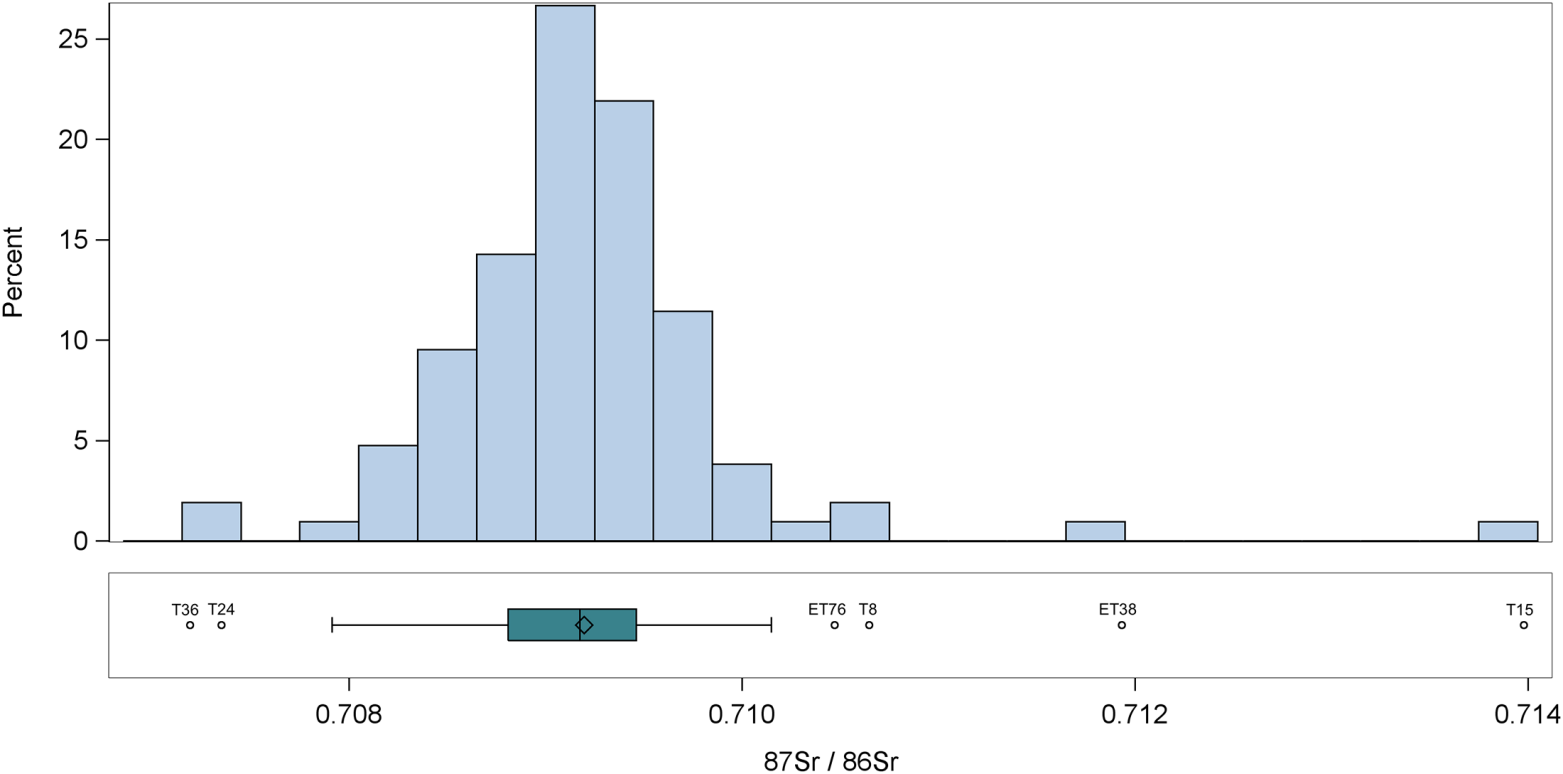
Strontium Isotope Ratios of All Imperial Period Individuals.

Strontium isotope data from both sites are plotted in Fig 3, a combination histogram and box plot. The histogram shows that the majority of the strontium isotope data points are between 0.708 and 0.710, a range consistent with the geology of the Colli Albani and with the remains of archaeological animals, and encompassing the contribution from rain and seawater, while the box plot identifies six possible outliers on the low and high ends: ET76 and ET38 from Castellaccio Europarco, and T36, T24, T8, and T15 from Casal Bertone.

Further interrogating the human data for outliers confirms many of these points. We applied the outlier labeling rule [117], which can detect outliers in normally distributed data through a calculation of quartiles. The outlier labeling rule resulted in lower and upper bounds, respectively, of 0.70701 and 0.71116. Individuals T15 and ET38 are both higher than the high bound, strongly suggesting they are from a geographical area other than Rome. We also used Grubb’s (extreme studentized deviate) test, which identified as outliers individuals T15 (z = 6.15; p <0.01), ET38 (z = 4.47; p <0.01), T36 (z = 3.46; p <0.05), and T24 (z = 3.39; p <0.05). While T15 and ET38 are clearly much higher in terms of their strontium isotope ratio than is expected from the rest of the population and incompatible with the local geology, T36 and T24 are lower than expected.

#### Local Oxygen Isotope Range and Results

A local oxygen isotope range for humans in Imperial Rome is similarly difficult to estimate, even given the fact that precipitation in the Italian peninsula has been well studied [118]. The Italian peninsula is bisected by the Apennine Mountains, and the average meteoric precipitation there has a lower mean *δ*^18^O value than do the east and west coasts. There are significant differences in mean environmental oxygen isotope ratios with altitude, as *δ*^18^O values decrease from the coast to the mountains. No significant differences exist, however, with latitude in peninsular Italy, such that *δ*^18^O values along the Tyrrhenian coast fall within the same isopleth. Longinelli and Selmo [118] report their measurement of Rome at -5.65‰, which falls within a mean annual *δ*^18^O value of meteoric water of −6 to −5‰ VSMOW along the west coast of Italy. As noted above, however, drinking and irrigation water in Rome came from local rainwater collected in cisterns, springs and groundwater in the Colli Albani, river water from the Aniene, and springs in the Apennine foothills near Subiaco, which rose to an elevation of about 440 m and lay about 70 km from Rome (see Fig 2). At the eastern aqueducts’ source, the mean annual *δ*^18^O value from precipitation is a full permil lower than at Rome [118].

Because of the effects of fractionation in the human body, because oxygen is sensitive to dietary differences among mammals, and because conversion equations introduce significant error, it is impossible to directly employ the available environmental or faunal measurements of *δ*^18^O values to create a human baseline for Rome [101, 108, 118]. Many researchers have therefore assumed that populations unaffected by immigration and trade or importation of food and drink will fall within an oxygen isotope range of about 2‰ [119], but there may be a trailing ‘tail’ on the positive end of a histogram of *δ*^18^O values as the result of importation of foodstuffs and/or boiling, evaporating, or brewing of water in the human diet [55]. Arriving at a proposed local oxygen isotope range therefore necessitates looking at variation within the Roman Imperial sample itself and comparing these data to those generated by Prowse and colleagues [76] at nearby Portus Romae.

**Fig 4:**
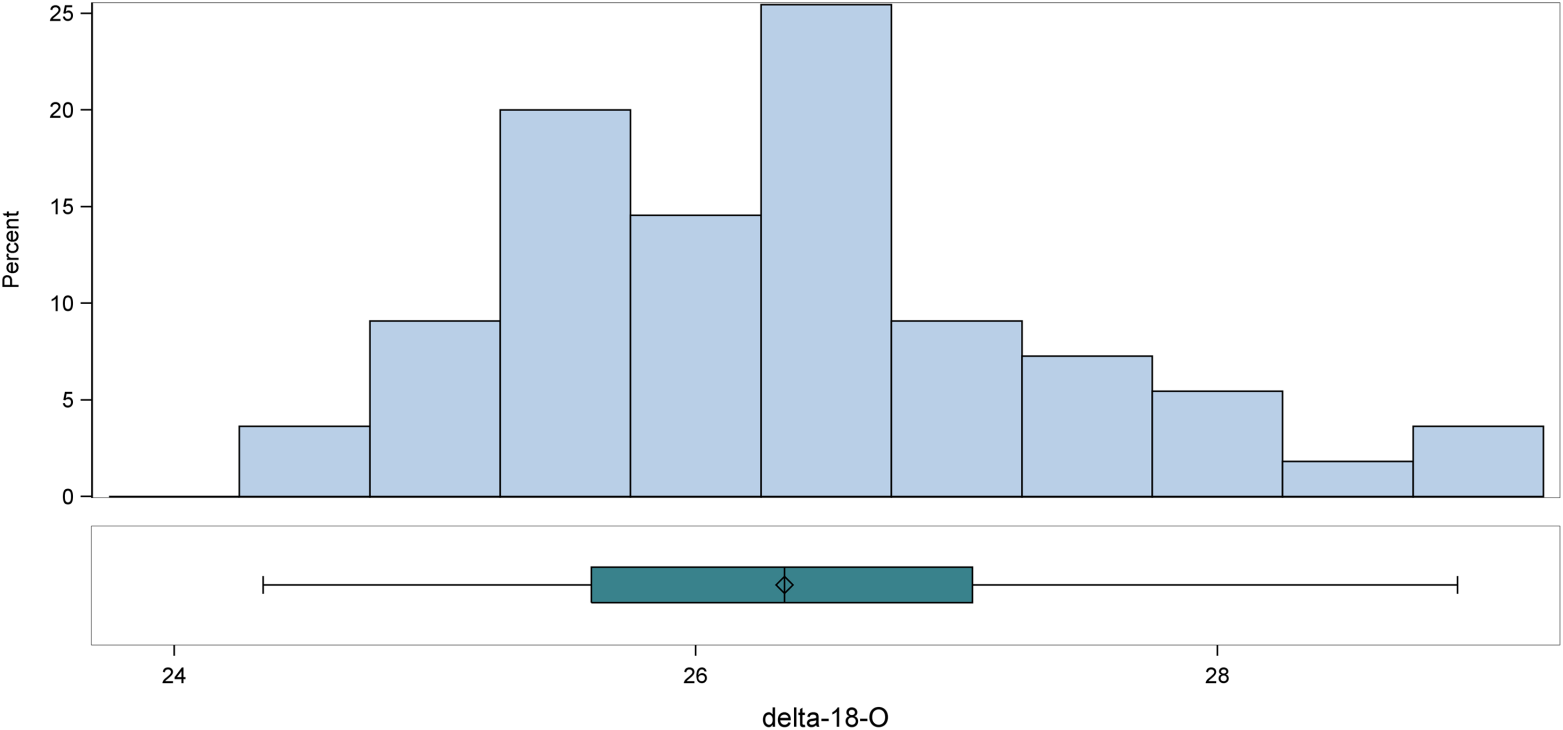
Oxygen Isotope Ratios of All Imperial Period Individuals.

For the entire Roman sample (n = 55) in Fig 4, *δ*^18^O values range from 24.3‰ to 28.9‰ VSMOW, for a total range of 4.6‰. If we allow for a 1‰ reduction due to imported aqueduct water and a 1‰ increase to allow for any influence of breastmilk consumption on the oxygen isotope ratios of these first molars, a 4‰ range is a conservative one for Imperial Rome. The mean *δ*^18^O values for the entire sample and for each site are tightly clustered. Over the whole sample (n = 55), the mean *δ*^18^O value is 26.3 ± 1.1‰ (1 stdev), and the site-specific means are not statistically different from this or from one another: Castellaccio Europarco (n = 14) is 26.5 ± 1.0‰ (1 stdev), while Casal Bertone (n = 41) is 26.3 ± 1.1‰ (1 stdev) (U = 253.5; p = 0.52; n = 55). The histogram and boxplot representations of these data show a continuous distribution with no outliers. Further interrogating the oxygen isotope data with the outlier labeling rule and Grubb’s test also produces no clear statistical outliers.

Putting oxygen isotope ratio measurements through a series of regression equations to see if they line up with expectations from meteoric precipitation is possible but must be done with caution. The *δ*^18^O_*carb*_ (VSMOW) values were first converted using [54] to *δ*^18^O_*phos*_ (VSMOW) values, then those values were converted using [55] to *δ*^18^O_*dw*_ (VSMOW) values. The mean *δ*^18^O_*dw*_ for Castellaccio Europarco (n = 14) is −6.3‰ ± 1.5 (VSMOW), while the mean *δ*^18^O_*dw*_ for Casal Bertone (n = 41) is −7.1‰ ± 1.6 (VSMOW).

Converting enamel *δ*^18^O to estimated *δ*^18^O of drinking water consumed during enamel formation shows that the majority of these data, in spite of the introduction of imprecision through conversion equations, are roughly in line with ratios recorded by [118] for Rome and its *suburbium* (−6 to −5‰ VSMOW), as well as its eastern aqueduct water sources (−7 to −6‰ VSMOW) [120]. Three individuals, however, are more than 2 stdev from the mean: T70, T36, and T39, all with higher than expected oxygen isotope ratios.

The results in this study also fall within the *δ*^18^O values previously obtained by Prowse and colleagues [76] from two samples: first molars of individuals buried in the Isola Sacra cemetery of Imperial-era Portus Romae and deciduous teeth from modern Roman children. Their study of *δ*^18^O values of the enamel of 20 deciduous teeth gathered from 15 modern children born in Rome yielded a mean of 26.0 ± 0.6‰ VSMOW (1 stdev). Note: The data from Prowse et al. 2007 [76] were published in the VPDB standard and have been convered here to the VSMOW standard using the equation 1.03092 * VPDB + 30.92 = VSMOW [121]. Further, their results from Isola Sacra (n = 60) give a mean of 25.4 ± 1.1‰ VSMOW (1 stdev).

**Fig 5:**
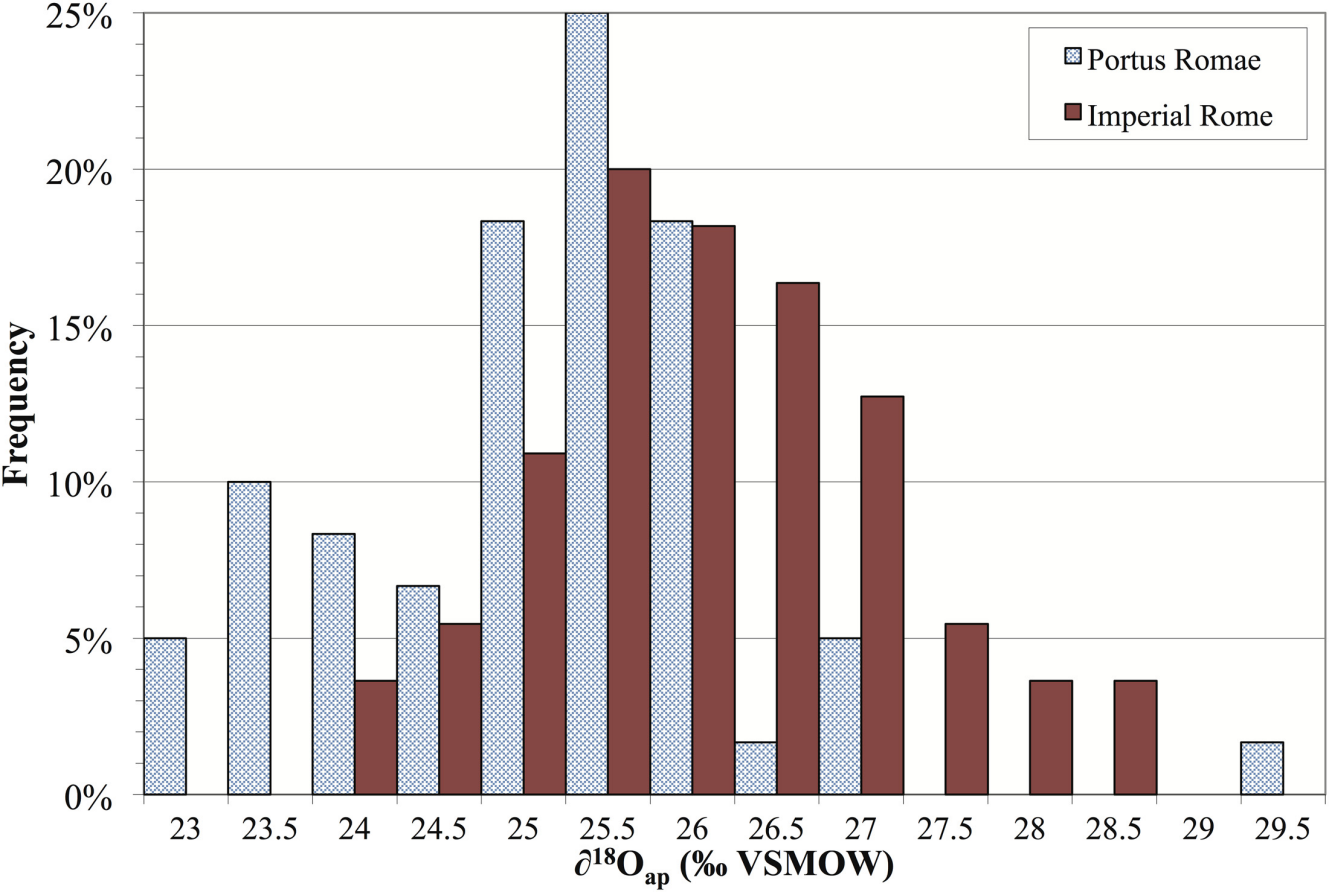
Histogram of Oxygen Isotope Ratios from Imperial Rome and from Portus Romae. Portus Romae data are from [76].

While the measured human *δ*^18^O values from Casal Bertone and Castellaccio Europarco overlap the Isola Sacra data, as evident in the histogram in Fig 5, the people buried at Isola Sacra tend towards lower *δ*^18^O values. All of these measurements come from first molars, which begin forming at birth and are complete around age 4. As Roman children were generally weaned between 6 months and 2 years [98, 122–126], differences in breastfeeding practices between Rome and Portus Romae could be evident in this case. However, the sampling protocol used in the current study took enamel from the tooth wall rather than from the earlier forming cuspal regions. Another potential cause for difference is in the water source itself, as inhabitants of Rome would have had greater access to aqueduct water than would inhabitants of Portus Romae; in this case, though, we would expect Romans to have lower *δ*^18^O values on average. Finally, it is possible that the wide range of oxgyen ratios is masking immigrants at either or both sites.

Oxygen isotopes of first molars alone are therefore incapable at the current time of clearly distinguishing between immigrants and locals at Rome, but testing multiple teeth that form at different times, as Prowse and colleagues did [76], has potential for future studies.

### Nonlocals at Imperial Rome

Strontium and oxygen isotope systems are complementary on the Italian peninsula, as strontium tends to vary north-to-south [102], while oxygen varies east-to-west [118]. In order to better understand migration and mobility in Imperial Rome, strontium and oxygen isotope results were combined from all individuals who were tested for both (n = 55).

**Fig 6:**
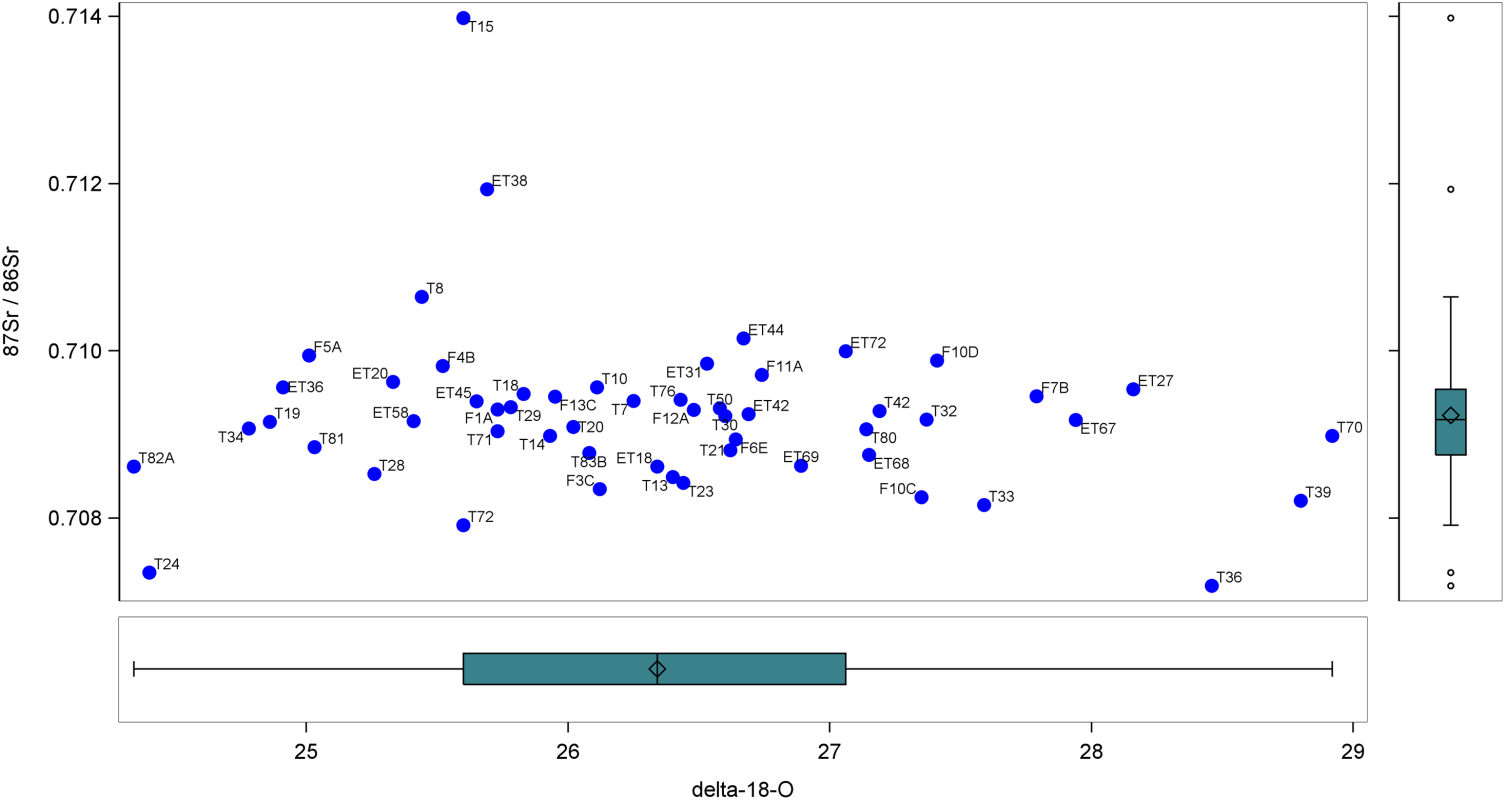
Strontium versus Oxygen Isotope Ratios from Imperial Rome.

Fig 6 is a scatterplot of each individual from Casal Bertone (n = 41) and Castellaccio Europarco (n = 14) who had a first molar tested for both strontium and oxygen isotopes. The wide range of oxygen isotope ratios continues to be evident, along with a clustering of strontium isotope ratios as noted above. A box plot along the oxygen axis reveals no outliers in this subsample of the population, while the box plot of the strontium isotope ratios again shows T15, ET38, T24, and T36 as statistically outside of the expected range of values.

**Fig 7:**
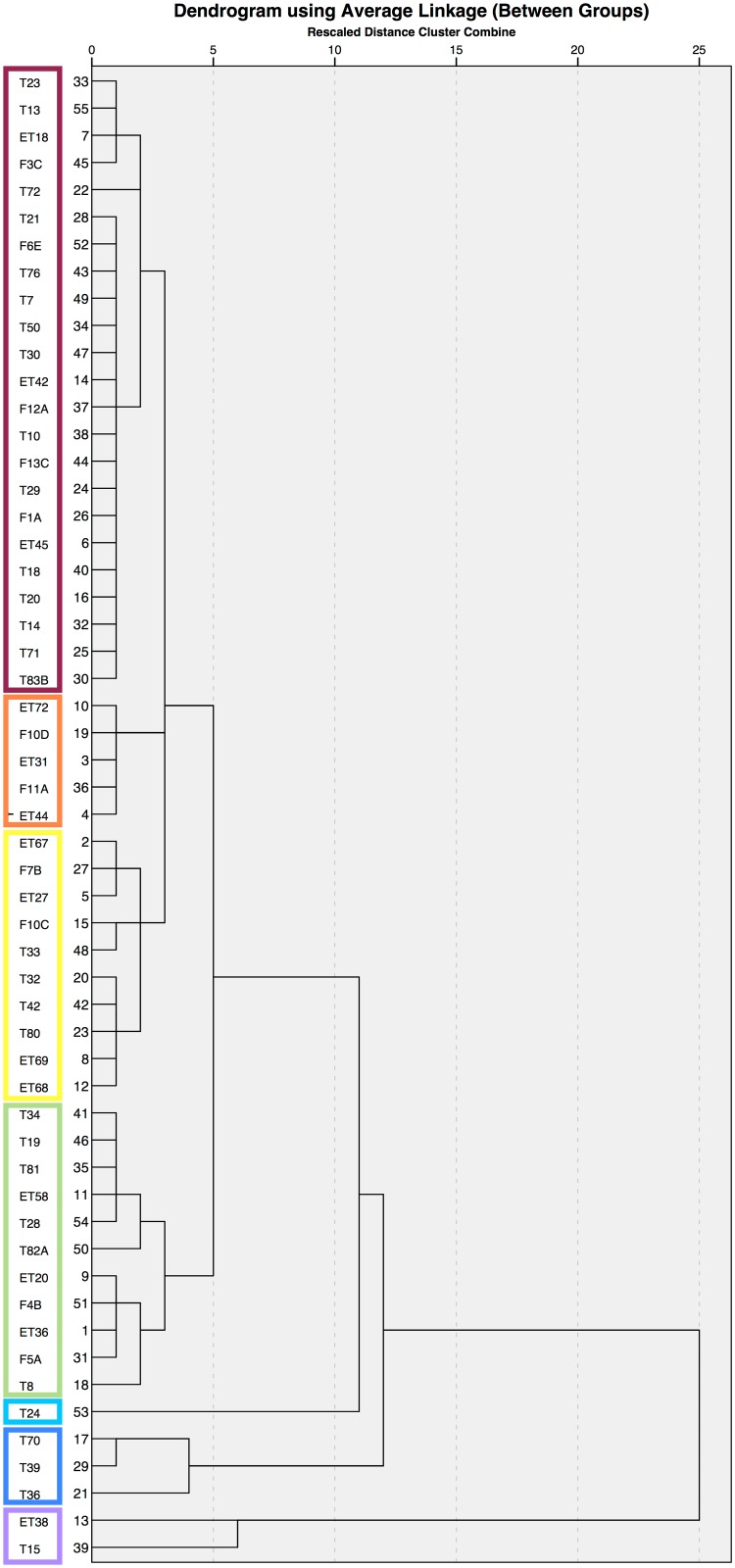
Hierarchical Cluster Analysis Results. Dendrogram shows average linkage based on Sr and O isotopic parameters for individuals with both (n = 55). Boxes represent the seven clusters that displayed the highest statistical significance using one-way ANOVA.

Multivariate statistical analysis can be performed on these ^87^Sr/^86^Sr and *δ*^18^O data to look for commonalities in groups [127]. Because of the small sample sizes, hierarchical cluster analysis is an appropriate method to examine the data for groups that may suggest common geographical backgrounds. The strontium and oxygen isotope ratios were first transformed into z-scores, as the scales for the two isotopes vary considerably. The analysis was run for models that imposed two, three, four, five, six, seven, eight, and ten clusters. A one-way ANOVA run on each model to identify the number of clusters that were most significantly different based on both isotopic parameters resulted in a model with seven clusters (strontium: F = 22.163, p = 0.000; oxygen: F = 39.224, p = 0.000) (Fig 7).

The three clusters that join late are the most interesting when compared with the scatterplot in Fig 6. Individual T24 forms a cluster, and this person has low strontium and low oxygen isotope ratios. Individuals T15 and T36 form a cluster, and both have average oxygen but very high strontium isotope ratios. Finally, the group with T70, T39, and T36 have high oxgyen and average-to-low strontium isotope ratios.

The most likely nonlocals, given all the data cited above, are T15, ET38, T36, and T24. The high oxygen isotope ratios of T70 and T39 may also mean they were immigrants, although this is not statistically conclusive, and the high strontium isotope ratios of T8 and ET76, similarly not statistically conclusive, could place these individuals as immigrants as well. Additional testing on different teeth from the same individual or with different isotopes or DNA analysis could bring more clarity to the geographical origins of this second set of potential immigrants.

## Discussion

The data generated in this study are useful for answering questions raised by Roman history, or more specifically by the lack of records of migrants to Rome. It is possible to start answering questions about demography, homeland, and acculturation using biochemical data drawn from human skeletal remains from Imperial Rome, in spite of the complexity of the environment that supported this ancient civilization. Nevertheless, additional work is needed to build on and refine these results.

### Who immigrated to Rome?

Of the nonlocal sample (n = 4), there are three adult males and one adolescent of unknown sex. Two of the males fall in the Middle Adult category (35-50) and one into the Older Adult category (50+), while the Adolescent is between 11-15 years old. The other four individuals whose isotope ratios were different from local Roman expectations, although not statistically conclusive, include two Older Children (7-12 years old), one probably male older Adolescent (11-15 years old), and one older Adolescent female (16-20 years old).

While it is not possible to tell when during their lives the adults came to Rome, the subadults provide a *terminus ante quem* of their age-at-death. The youngest possible nonlocals were found in the Older Child category, suggesting these individuals came to Rome between the time their first molar crown finished forming (age 4) and their deaths (before age 10). Although migration of children is not well known in the historical record, Prowse and colleagues [76] similarly found nonlocals among their subadult sample from the Isola Sacra cemetery.

The clearest evidence for migrants to Rome from these two archaeological skeletal collections is within the male population, with possible evidence of migration of children and a woman as well. As noted above, however, females are underrepresented in particular at Castellaccio Europarco, so this may not reflect the real sex ratio of immigrants. Whereas Prowse and colleagues [76] suggested their finding of migrant children at Portus Romae was related to family migration, it is unclear whether women and children immigrated individually or as part of a male-headed family to Portus [128, 129] or to Rome, and there is no evidence from any of these Imperial cemeteries of family groups. As noted above, however, females are underrepresented in the two cemetery populations, which may be contributing to a bias in favor of males as anomalous individuals.

It is also impossible to answer from the present data whether these individuals were voluntary or compulsory migrants. The status of slave was multifaceted and mutable during the Empire [130], and there is no indication in the archaeological information from Casal Bertone and Castellaccio Europarco that any specific individual was a slave. There is, however, no evidence from isotopes that individuals buried in the mausoleum at Casal Bertone were nonlocal, whereas the necropoleis at Casal Bertone and Castellaccio Europarco both produced skeletons with nonlocal isotope ratios. Burial in a necropolis was customary for the lower classes, while burial in a mausoleum cost more [77]. These isotope data may be showing a form of economic, status-related migration, with more lower class individuals and possibly slaves moving to Rome compared to wealthier individuals. Additional testing would be needed, though, to confirm this hypothesis.

### Where did immigrants come from?

Because migrants often came to Rome in diasporic waves resulting from slavery, attempting to identify a general geographic origin can be instructive. The combination of strontium and oxygen isotope analyses is particularly useful for this in western Europe, although only general predictions of homeland can be made. Oxygen isotopes on the continent vary roughly east-to-west, while strontium isotopes are higher in the older rock of mountains such as the Alps and lower in the younger rock of volcanic areas like most of peninsular Italy. From the perspective of Rome, oxygen isotope ratios will decrease as one moves into the Apennine range running along the spine of Italy, and strontium isotope ratios will increase to the north and decrease to the south.

The four individuals with clearly anomalous isotope ratios—T15, ET38, T24, and T36—fall into three distinct strontium and oxygen isotope combinations. T15 and ET38 have oxygen isotope ratios within range of Rome, but strontium isotope ratios that are significantly higher, suggesting a possible origin in a place with older geology, such as the Alps or one of the islands in the Tyrrhenian Sea. As people arrived at Rome from all over the Empire, however, there are numerous locations in which these individuals could have been born.

Individual T24 has low strontium and low oxygen isotope ratios compared to Rome, suggesting an origin somewhere with a cool, wet climate and basalt or limestone substrate, such as the Apennines. Individual T36 has high oxygen and low strontium isotope ratios, suggesting an origin in a region of limestone or basalt with a hotter, drier climate than Rome, such as North Africa. For these individuals, however, a dietary explanation for the anomalous strontium isotope ratios, while much less likely owing to the concomitant *δ*^18^O values, cannot be completely ruled out. As Rome imported significant amounts of grain from north Africa during the Empire, and as human strontium isotope ratios from Egypt and the Nile Valley have been shown to be lower than those in Rome (around 0.707 to 0.708) [131], it is not impossible that T24 and T36 were consuming a significant amount of imported grain as children. Still, as shown further below, the dietary explanation is less likely than is an origin elsewhere.

The four additional individuals whose isotopes may indicate they were immigrants—T8, T70, T39, and ET76—fall into the categories above. T8 and ET76 have higher-than-expected strontium isotope ratios, showing up as outliers in the box plot in Fig 3. They may have arrived at Rome from a region of older geology such as northern Italy. Individuals T70 and T39, while not statistical outliers in the oxygen isotope box plot in Fig 5, are nevertheless 0.6-0.7‰ higher than the next closest local, suggesting they may also be immigrants. They could have arrived at Rome from a drier climate like North Africa. These four individuals highlight the challenge of identifying immigrants to Rome from a vast geographical expanse.

Finally, the fact that there is a large spread in both the strontium and oxygen isotope data compared to results obtained from other archaeological populations could indicate that people were arriving at Rome from places not too far removed, in a form of centripetal migration, as Prowse and colleagues [76] suggest for Portus. Both the strontium and the oxygen isotope ratios from Rome are diverse, and it is not unreasonable to assume that these may reflect the diversity of the population as well. It is also possible that even more individuals are essentially isotopically invisible migrants, if they came to Rome from homelands with similar strontium and/or oxygen isotope values. Further isotopic and DNA work will be necessary to better understand origins and homelands from skeletal remains.

### Did immigrants acculturate after their arrival?

In a study of mobility and identity in Bronze Age south-central Italy, M.A. Tafuri [132] looked at foodways as a potential characteristic by which people may have expressed their individual or collective identities. Tafuri concluded from her trace element analysis that the anomalous diets of females from the site of Sant’Abbondio represented a way of expressing their identity or *habitus* in spite of their post-marital residence change. She writes that, “in creating the link between place and resources, between living and eating, the essence of individual and social identity can be re-created” [132].

**Fig 8:**
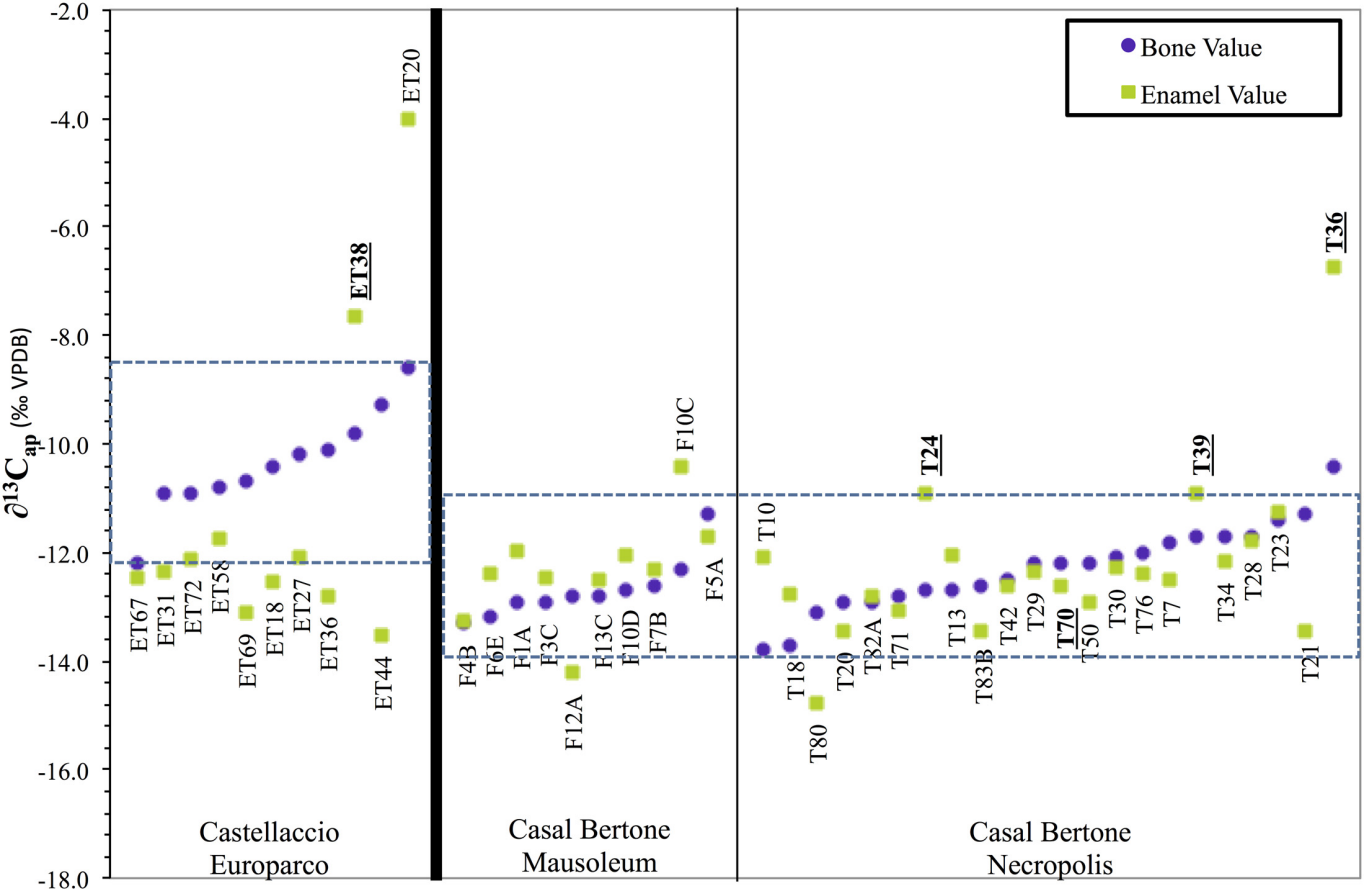
Enamel and Bone *δ*^13^C_*ap*_ Measurements. Bold, underlined sample IDs indicate individuals identified as immigrants to Rome. Dashed line represents the 2 stdev range of bone isotope ratios for each archaeological population.

With few grave goods and no anomalous burial styles at either Casal Bertone or Castellaccio Europarco, it is largely unclear whether the immigrants to Rome are similar to the locals because of a choice to portray themselves as locals or because of a simple lack of resources to differentiate themselves. The carbon isotope data hint at a form of acculturation, however, and provide another line of evidence of nonlocal origins. In this study, *δ*^13^C_*ap*_ values were measured from enamel and compared with previously published bone values [98]. Comparing these two data points for each individual provides the opportunity to look at changes that may have occurred in the diet between childhood (0-4 from the first molar crown) and the years leading up to death (bone sample). Fig 8 displays these data for the 43 individuals who were subjected to analyses for ^87^Sr/^86^Sr, *δ*^18^O, and *δ*^13^C_*ap*_ of bone and enamel.

Killgrove and Tykot [98] showed that the *δ*^13^C_*ap*_ values from bone, when combined with *δ*^13^C and *δ*^15^N, reflect a diet composed of wheat with variable inputs from legumes, meat, and seafood. The entire population save one individual (ET20) has a C_3_ protein signature based on the plot of *δ*^13^C_*co*_ and *δ*^13^C_*ap*_ coupled with low *δ*^15^N. Individual ET20, who has a very high *δ*^13^C value, nevertheless has *δ*^13^C_*ap*_ and *δ*^15^N values in line with the rest of the population, suggesting consumption of millet rather than seafood. The higher *δ*^13^C_*ap*_ bone values for Castellaccio Europarco therefore likely reflect greater reliance on millet for this suburban population compared to the periurban population of Casal Bertone [98]. Construction of a 2 stdev range for bone *δ*^13^C_*ap*_ values for each site yields −12.2 to −8.5‰ VPDB for Castellaccio Europarco and −13.9 to −10.9‰ VPDB for Casal Bertone. Individuals whose *δ*^13^C_*ap*_ ratios fall outside that range may have been eating food of nonlocal origin.

A pattern therefore emerges in which individuals identified as nonlocal to Rome based on strontium and oxygen isotope ratios are most likely to have *δ*^13^C_*ap*_ enamel ratios outside the 2 stdev local range but bone ratios within it. In particular, individuals ET38, T24, T39, and T36 have anomalous strontium and/or oxygen isotope ratios, higher enamel carbon isotope ratios than expected for Rome, but also bone isotope ratios in line with the local range. While many people changed their diets between the time they were children and the time they died—most notably at Castellaccio Europarco, where the adult diet likely contained significant quantities of millet or millet-foddered animals [98]—those people with suspected nonlocal origins changed their diets more dramatically, likely due to a difference in available foodstuffs at Rome compared to their geographical area of origin. Whether this change was voluntary (to fit in with Roman foodways) or involuntary (because of food availability) is not clear.

### Conclusions

Modeling migration to Imperial Rome is necessary for a deeper understanding of demographics, family structure, and gender roles, and is particularly relevant for the vast majority of the Roman population that was left out of historical records. This study has generated the first concrete data of individuals who were not born at Rome, but much more research is needed into a variety of data sets to fully contextualize questions about mobility in Imperial Rome and to move forward in employing bioarchaeology in Roman migration studies.

Chemical analysis of small animals could help define the local range of bioavailable strontium in a volcanic area with quite complex geology, contributing to our ability to identify both immigrants to Rome and their homelands. Further studies of strontium and oxygen isotopes of ancient Romans would similarly contribute to the understanding of the use of aqueduct water, whose importance in Roman culture and to Roman health cannot be overstated. Analyses of multiple teeth from a single individual would greatly aid our assessment of mobility and potentially allow us to characterize migration as rural-to-urban, urban-to-rural, or circular [76]. Further, multiple isotopes are needed to better understand the complex society of Imperial Rome. Strontium and oxygen isotope values do not fully capture the individuals who migrated to Rome or the variation among them, so the addition of sulphur and lead isotopes (see also [133]) may clarify the picture of migration as may DNA analysis [134]. Additional avenues of research interest include looking at patterns in habitual action through enthesopathies or musculoskeletal markers as these may show, for example, distinct local and nonlocal patterns of leatherworking in a burial population. Spatial relationships among graves may also hold clues to the composition of polyethnic communities in Rome, particularly since burial could be based on shared occupation or ethnicity and financed through a *collegium* (fraternal organization), although preliminary GIS analyses at Casal Bertone did not reveal patterns in strontium isotope data suggestive of diaspora [135].

A bioarchaeological approach to Imperial Rome that combines ancient history, anthropological theory, material culture, and chemical analyses as outlined above will help reveal individual migrants, contextualize them within migrant and local populations, and elucidate the experiences migrants had at Rome and elsewhere in the Empire. Immigrants contributed physical labor, novel pathogens, and diverse genes to the city, and Rome in turn affected these individuals’ lifestyles and experiences through social, environmental, and economic conditions. Bioarchaeological studies that mesh palaeopathology and biochemical analysis may reveal additional facets of daily life and health outcomes for both immigrants and locals. For example, cribra orbitalia could be related to dietary deficiency, a pathogen-heavy environment, or lead poisoning, and a multi-element analysis of Pb, C, N, and O may tease out the reasons [136]. The thousands of individuals who died in Rome and whose skeletons survived two millennia of burial hold vast amounts of information about this civilization, and telling their stories through osteobiography will further complement, contradict, and complicate the received wisdom about the Roman Empire.

Finally, historically-informed bioarchaeology in Rome may be particularly fruitful in terms of generating a more complex theory of slavery in the Empire. While free, mobile individuals in Imperial Rome traveled, wrote letters, and sent money back home while living elsewhere [137, 138], many migrants arrived at their destination in Italy as slaves. Some of the immigrants identified in this study may have come to Rome as slaves, but their tenure as slaves could have been ended quickly by manumission. Although the bioarchaeology of slavery is even more fraught with issues than the bioarchaeology of immigrants, it remains an important future avenue of research because slaves comprised a considerable segment of the Roman population.

It is imperative, therefore, to assess migration at all levels in the Roman Empire in order to better understand the epidemiology and demography of the capital but also to proceed further with questions about the people of Imperial Rome. A contextually-informed historical bioarchaeology is necessary to bring ancient migration studies to the fore and to reveal the role that migrants had in shaping the history, culture, and geography of the Roman Empire.
